# Dental injury offenses and compensation imposed by Spanish courts, before and during Covid-19. A cross-sectional study

**DOI:** 10.1038/s41598-023-43863-8

**Published:** 2023-10-03

**Authors:** Ana Isabel Serrano, Javier Aragoneses, Ana Suárez, Cinthia Rodríguez, Juan Manuel Aragoneses

**Affiliations:** 1 Centro de Estudios Garrigues, Avenida Fernando Alonso nº 8, Alcobendas, Spain; 2 Department of Medicine and Medical Specialties, Faculty of Health Sciences, University of Alcalá, 28801 Madrid, Spain; 3 Department of Preclinical Dentistry, School of Biomedical Sciences, Universidad Europea de Madrid, Villaviciosa de Odón, 28670 Madrid, Spain; 4 Department of Dentistry, Universidad Federico Henriquez y Carvajal, 10106 Santo Domingo, Dominican Republic; 5 Dean of The Faculty of Dentistry, Universidad Alfonso X El Sabio, 28691 Madrid, Spain

**Keywords:** Dentistry, Health care economics

## Abstract

Injuries to the dentofacial region caused by third parties can affect physiological, sensory and esthetic functions with legal repercussions. The personal and social circumstances generated by Covid-19 and the governmental measures taken to control it, have increased the risk factors for violence and with it, the resulting injury rate. The aim of the present investigation was to compare the amount of civil liability for dental injury crimes agreed by Spanish courts, in certain Autonomous Communities, before and during the pandemic situation caused by Covid-19. For this purpose, a analytic cross-sectional study was carried out by analyzing sentences from the database of the Judicial Documentation Center. A comparison of means (one-way ANOVA) was used on the amount of compensation between the different years, and between the Autonomous Communities of Madrid, Catalonia Cataluña, Andalusia, the Canary Islands and the Valencian Community. It was observed that the year 2020 stood out for the increase in the number of cases of dental injury offenses. For its part, the Autonomous Community of Andalusia showed the highest amount of compensation during the pandemic, although the highest number of cases corresponded to the Community of Madrid. The statistical analysis yielded a probability of more than 0.05, which eliminated the possibility of significant differences in each of the comparisons.

## Introduction

Currently, the world panorama shows an increase in violent acts that affect a significant number of people regardless of age, gender or social position. Likewise, the incidence of these crimes can vary from one continent to another and show significant differences within the same region^1^.

From a medico-legal point of view, injury crimes are of significant importance, not only because of the double treatment they deserve, but also because of the important casuistry they represent in Spanish courts and tribunals, and because of the seriousness of the attack on physical integrity they represent^2^.

According to data from the European Statistical Office (Eurostat), Spain is considered one of the safest countries. However, when examining the categories of violent crime, it can be seen that assault in our country occupies an intermediate position with a rate of 37.2 per 100,000 inhabitants^3^. It should be noted that criminal proceedings and compensation are more frequent, as these criminal acts undermine people's quality of life and, consequently, their health^4^.

In this sense, dentistry as a science plays a decisive role not only in the identification of lesions affecting the oral cavity, but also in their diagnosis^5,6^. It is also a valuable and essential tool for the justice system in criminal proceedings for dentofacial injuries, and in the issuance of opinions and amounts of civil liability (CL)^7–9^.

While many dental injuries are the result of everyday accidents, others require a judicial investigation: injuries caused by third parties (assaults, fights, child abuse, domestic violence), accidental injuries or injuries in the workplace^6,10–12^.

Injuries in the oral area rarely cause permanent disabilities, due to their own nature and the improvement of restorative methods. However, in specific cases where the injury is associated with maxillofacial damage, or in individuals who develop professions in which the stomatognathic apparatus is fundamental to their practice, the consequences may persist over time^13^.

At the individual level, dental lesions increase with the development of modern life. Thus, they generate functional and esthetic problems, loss of income due to recovery time and, in general, a negative influence on the well-being and social relationships of those who experience them^14,15^.

In addition, they are characterized by being complex and diverse in terms of extent, tissues or anatomical areas affected^6^. Adequate examination and recording are aspects of vital importance, as the dentist is increasingly required as an expert to assess the repercussions of dentofacial damage.

Consequently, when faced with the action of a traumatic agent, the lesions affecting the dentofacial complex can be localized in different anatomical areas.

These include soft tissue injuries (cheeks, lips, tongue, skin involvement, mucous membranes, muscles, vessels, nerves and glands) characterized by ecchymosis, hematomas, erosions, abrasions, wounds, puncture, cutting, contusions and contusions. There are also lesions in bone tissues (mandibular and maxillary bone), which are fissures and fractures^10^.

Also, injuries to the temporomandibular joint (TMJ) can be identified, recognized by contusions, dislocations, fractures of the condyle and glenoid fossa. As for dentoalveolar lesions in the supporting tissues, concussion, subluxation, luxation and avulsion can be distinguished. Finally, there are injuries to the dental hard tissues, represented by enamel, coronary, corono-radicular and radicular fractures^10^.

The correct evaluation of damage and possible sequelae is essential to determine the lesions that can be restored, the esthetic damage and those that will be permanent^13^.

In addition to the above considerations, national or international crises, emergencies and times of unrest are associated with an increase in interpersonal violence. Pandemics are no exception, and in the wake of the COVID-19 epidemic, it is necessary to evaluate the incidence of the problem in Spain^16^.

In this context, SARS-CoV-2 caused severe acute respiratory syndrome (SARS-CoV-2) had a public health impact on the international community^17–19^. Radical measures, such as confinement, proved to be the most effective way to reduce contagion^20^. However, the World Health Organization (WHO) called attention to the creation of increased family contact which, while restricting transmission of the virus, is prone to increased violence and with it, the rate of injury crime^21^.

Spain recognizes the vulnerability to which women and children are exposed during confinement. On the other hand, there is a decrease in mortality and complaints of physical injury, although subsequently, an increase is observed during the reopening and return to the new normality^22^.

The main objective of this study was to analyze the number of dentofacial lesions during the Covid-19 period in the different Autonomous Communities of Spain, in order to provide courts and tribunals with the necessary information to make informed decisions regarding the imposition of penalties and the determination of compensation amounts, by comparing the casuistry of these crimes and the compensation awarded in different regions, both before and during the Covid-19 pandemic.

The null hypothesis was: there are no differences in the number of dental injuries, as well as between the means of the compensation amounts in the pre-Covid-19 period and in the Covid-19 period among the different Autonomous Communities. The alternative hypothesis was: there are differences between the means of the amounts and the number of dental injuries.

## Materials and method

This analytic cross-sectional study followed the STROBE checklist to ensure proper presentation of the results and methodology used. This study was carried out in accordance with the ethical principles laid down in the Declaration of Helsinki. The research was based on the collection of information using the database of the General Council of the Judiciary of Spain, known as CENDOJ. It is important to emphasize that all the judgments included in CENDOJ are freely and publicly accessible. This fundamental characteristic of the data source used ensures that the principles of accessibility, transparency and equity are respected in this research.

From the total database of the Judicial Documentation Center and ordered by Autonomous Communities, which consisted of 199 resolutions, 9 had to be discarded, since they corresponded to centralized judicial bodies that did not represent territorially the place where they took place. Of the remaining 190, the following Communities were selected: Madrid, Catalonia, Andalusia, Canary Islands and Valencia, due to the greater number of incidents registered in these Autonomous Communities, in comparison with the remaining national ones in the period under review.

Therefore, the population under study consisted of all the resolutions issued in the corresponding Autonomous Communities (190) for dental injury offenses during the period from January 2019 to May 2021.

The variables studied were the total number of dental injuries by Autonomous Community and year, as well as the amount of penalties in euros.

The statistical program SPSS version 25.0 for Windows was used to process the information. Descriptive statistics allowed the summary of the information with measures such as the mean and standard deviation for continuous variables, and the percentage for categorical variables.

To compare the amount of indemnities, both between the different years and between Autonomous Communities, a comparison of means (one-way ANOVA) was used. We worked with a reliability of 95% in all hypothesis tests. The results are illustrated in text, tables and statistical graphs.

## Results

Table 1 shows the sentences for injury crimes in the dental field, according to year of application and amount in euros. It was possible to observe an increase in the total amount in 2020 with respect to 2019, and it is important to note that, although the year 2021 shows lower values, we only had access to information for the first quarter of the year, clearly observing the trend towards an increase in the amount of compensation, so it can be inferred that this, at the end of 2021, may be above the previous years. The statistical technique yielded a probability associated with the test statistic greater than 0.05, so there is insufficient evidence to suggest a significant difference between the means of the amount in the three years, which, in the authors' opinion, is closely related to the above.

**Table 1:** Sentences for injury crimes in the dental area according to year of application and amount civil liability euros. Source: Sanctions Register of the Judicial Documentation Center. Anova F = 0.188, *p* = *0.829.*

Year	Amount	Ẋ	of	95% confidence interval	
				Lower limit	Upper limit
2019	470,536	7842.26	15,696.88	3787.33	11,897.20
2020	560,882.38	7579.49	9821.42	5304.05	9854.93
2021^a^	362,653.25	6475.95	12,714.55	3070.97	9880.93

^a^Dates January–May.

**Figure 1:**
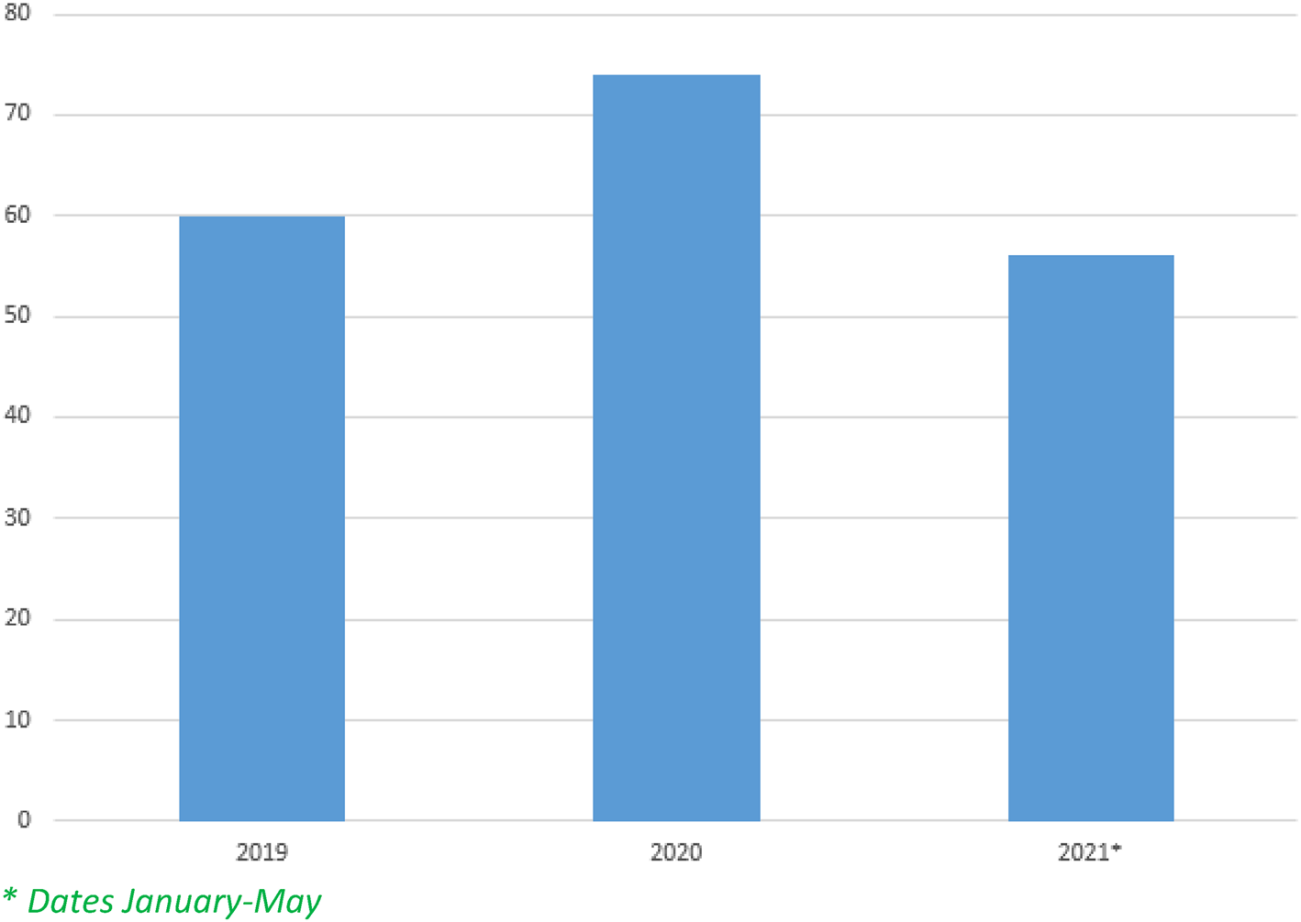
Sentences for dental injury crimes by year. *Dates January–May*.*

Figure 1 corroborates the above criteria and illustrates an increase in the number of sentences handed down during 2020. For its part, the first quarter of 2021 is close to the previous ones.

Table 2 shows that the highest amount of compensation ordered in 2019 by the judicial bodies of the Autonomous Communities studied corresponded to Catalonia, followed by the Autonomous Community of Madrid. On the other hand, it is observed that the highest amount of compensation during 2020 and the first quarter of 2021, belonged to Andalusia. The statistical analysis performed showed a significance greater than 0.05 for the differences in the means, a result that is considered insufficient to confirm a statistically significant difference between the amount of these Autonomous Communities in the different years.

**Table 2:** Sentences for dental injury crimes, by year of application Autonomous Community and amount CL euros. Source: Sanctions Register of the Judicial Documentation Center. Anova F = 2.046 *p* = *0.074.*

Year	Autonomous	Amount	Ẋ	of	95% confidence interval	
					Lower limit	Upper limit
2019	Madrid	40,684.78	2393.22	3114.69	791.79	3994.65
	Andalusia	5912.52	1970.84	2062.53	3152.76	7094.44
	Catalonia	234,966.86	26,107.42	32,300.09	1279.38	50,935.47
	Canary Islands	61,644.27	8806.32	13,976.80	4120.06	21,732.71
	Valencia	10,900.00	2180.00	1571.37	228.89	4131.11
2020	Madrid	103,956.56	6115.10	7439.46	2290.08	9940.11
	Andalusia	119,390.31	10,853.66	14,230.96	1293.18	20,414.15
	Catalonia	51,634.05	6454.26	10,581.16	2391.81	15,300.33
	Canary Islands	73,888.34	8209.82	8116.38	1971.01	14,448.62
	Valencia	49,952.87	6244.11	6537.70	778.46	11,709.76
2021	Madrid	20,042.04	1670.17	3288.61	1419.31	3759.65
	Andalusia	273,614.5	3506.33	4494.69	1210.56	8223.22
	Catalonia	16,131.05	3226.21	5279.93	3329.69	9782.11
	Canary Islands	26,266.04	13,133.02	9711.38	74,120.23	100,386.27
	Valencia	62,214.05	20,738.02	35,919.30	68,490.47	109,966.5

**Figure 2:**
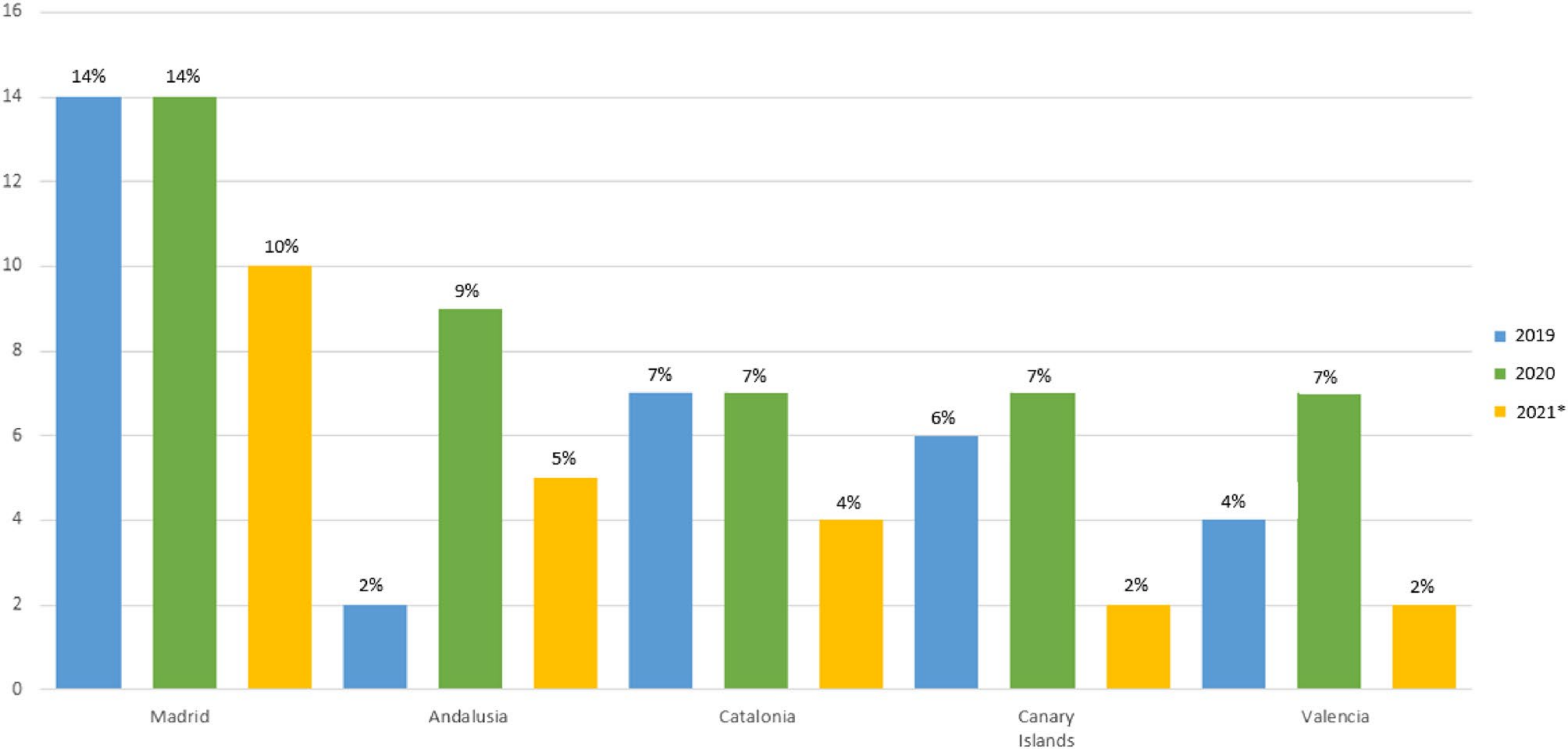
Sentences for dental injury crimes by Autonomous Community and year. *Dates January–May*.*

Figure 2 shows the percentage of sentences for dental injury crimes according to the main Autonomous Communities and years. It can be seen that the highest number of sentences handed down in the period analyzed corresponds to the Autonomous Community of Madrid. It is important to note that this result is independent of the amount of compensation, as the latter varies according to the type and degree of injury.

## Discussion

The results of the research show how Spain and some of its Autonomous Communities have suffered the effects of Covid-19 in the field of violence, by showing an increase in the number of judicial resolutions for crimes of dental injuries and compensation during 2020, as a result of these acts.

Likewise, the rise in sentences in 2021 confirms that the pandemic context deteriorates the situation of dentofacial health, and generates an increase in cases of violent acts, product of confinement^23–25^.

Statistics shown in other researches^10,14,26^ coincide with the results of the present article, regarding the gradual increase of dental injuries in recent years (39% of sentences for dental injury crimes in 2020 which is higher than 32% in 2019).

At the same time, interpersonal injuries in the dentofacial area are recognized as one of the main ones in developed countries, corresponding to 26.6% in men, and 11.6% in women^27^.

Some authors, such as Esses et al.^11^ in a study conducted in Brazil, observe that the main causes of oral injuries are, in the first place, traffic accidents (54.1%) and, in second place, aggressions (20.1%). These findings are contrasted by the studies of Morales et al.^28^, where the main mechanism of action was aggression by third parties (52%), followed by automobile accidents (18.1%), and motorcycle accidents (17.8%).

It is noted that the Courts and Tribunals, both nationally and in the Autonomous Communities of Madrid, Catalonia, Andalusia, the Canary Islands and the Valencian Community, did not cease proceedings for dental injury offenses during the pandemic. The amounts of the civil liability ruled by the same exceeded the compensations of the cases prosecuted in 2019.

Within these Communities, Madrid stands out, which, despite mobility restrictions, social distancing and quarantine, during 2020 equaled the figure of the previous year with 17 cases prosecuted. For its part, the first quarter of 2021 appears with a gradual rise in crimes.

In this same context, the Andalusia Autonomous Community shows the highest amount of compensation in only 15 months. Gender violence is increasing in the midst of the health emergency in Spain, and illustrates the case of 44 women who, together with 47 minors under their custody, were admitted to the shelters of the Andalusia´s Institute for Women, during the first month of home confinement after the decree of the state of alarm^29^.

The increase in these crimes in Spain is correlated with the data provided by the United Nations (UN) published at the end of September 2020, which shows an increase of 25% in Argentina, 30% in Cyprus and France, and 33% in Singapore, among others^30^. Mexico is one of the Latin American countries most affected in this regard^31,32^.

This shows that the pandemic and prevention measures do not constitute a barrier to these crimes^16^. Thus, it is corroborated that sociocultural factors are a consequence of the interactions of a group and not of a subject per se^20^. However, it should be noted that at the time of this study data was available from 2019 to May 2021.

## Conclusions

The dentist is most frequently requested to assess, as an expert, the importance and repercussion of dentofacial lesions. These lesions have a variable behavior and constitute a dental emergency due to their high incidence. They often affect soft tissues as well as dental and supporting tissues.

The pandemic caused by Covid-19 has not only been a health crisis for Spain and the rest of the world, but has also had an impact on criminality. The measures to contain it did not constitute an obstacle to the commission of criminal acts such as assaults or violent acts in their various variants.

The number of cases prosecuted during 2020 are higher than the records of 2019, with a tendency to an increase in 2021. Madrid was evidenced as the Autonomous Community with more cases registered, before and during the Covid-19 pandemic. The amount of indemnities highlighted Andalusia -as the most affected Autonomous Community.

## Data Availability

The data and material are available from the corresponding author.
